# Health risk factors in Australian Stroke Survivors: A latent class analysis

**DOI:** 10.1002/hpja.706

**Published:** 2023-02-28

**Authors:** Brigid Clancy, Billie Bonevski, Coralie English, Robin Callister, Amanda L. Baker, Clare Collins, Michael Pollack, Parker Magin, Alyna Turner, Jack Faulkner, Ashleigh Guillaumier

**Affiliations:** ^1^ School of Medicine and Public Health, College of Health, Medicine and Wellbeing, The University of Newcastle Callaghan NSW Australia; ^2^ Hunter Medical Research Institute, John Hunter Hospital New Lambton Heights NSW Australia; ^3^ Flinders Health and Medical Research Institute, College of Medicine and Public Health, Flinders University Bedford Park SA Australia; ^4^ School of Health Sciences, College of Health Medicine and Wellbeing, The University of Newcastle Callaghan NSW Australia; ^5^ Hunter New England Local Health District, John Hunter Hospital New Lambton Heights NSW Australia; ^6^ School of Biomedical Sciences and Pharmacy, College of Health, Medicine and Wellbeing, The University of Newcastle Callaghan NSW Australia; ^7^ IMPACT Strategic Research Centre, School of Medicine, Deakin University Geelong Vic. Australia

**Keywords:** alcohol drinking, health risk behaviours, latent class analysis, recurrent stroke, risk factors, secondary prevention, stroke

## Abstract

**Issues Addressed:**

To (i) determine the prevalence of health risk factors (physical activity, diet, alcohol, smoking, blood pressure medication use and mental health) in community‐dwelling stroke survivors; and (ii) examine how these health risk factors cluster, and identify associations with physical functioning, independent living, or sociodemographic factors.

**Methods:**

A secondary analysis of data obtained during a national randomised controlled trial. Participants had experienced stroke and completed a baseline telephone survey on demographic and stroke characteristics, health risk factors, physical functioning and independence in activities of daily living. A latent class analysis was performed to determine health risk profiles. Univariate logistic regressions were performed to identify if participant characteristics were associated with resulting classes.

**Results:**

Data analysed from 399 participants. Two classes of health risk factors were identified: Low Mood, Food & Moves Risk (16% of participants) and Alcohol Use Risk (84% of participants). The Low Mood, Food & Moves Risk group had poorer diet quality, lower physical activity levels and higher levels of depression and anxiety. Lower levels of independence and physical functioning were predictor variables for this group. In contrast, the Alcohol Use Risk group had better physical activity and diet scores, significantly lower probability of depression and anxiety, but a higher probability of risky drinking.

**Conclusions:**

We identified two distinct health risk factor groups in our population.

**So What?:**

Future interventions may benefit from targeting the specific needs and requirements of people who have experienced stroke based on their distinct risk group. Alcohol consumption in poststroke populations requires further attention.

## 1 INTRODUCTION

Globally, one in four people aged over 25 will have a stroke in their lifetime,^1^ but the mortality rate has been decreasing.^2^ As a result, there are more than 80 million people who have experienced a stroke living worldwide^2^ and they are living with an increased risk of experiencing another stroke.^3^ Health risk factors such as diet, physical activity, smoking status, alcohol consumption, mental health and blood pressure play an important role in the prevention of recurrent stroke.^4^, ^5^, ^6^ With recurrent strokes carrying a higher risk of morbidity and mortality,^7^ reducing the risk of recurrent stroke for each stroke survivor is vitally important.

Many people who have experienced stroke participate in and experience health risk factors that put them at increased risk of recurrent stroke. Most stroke survivors do not meet recommended guidelines for physical activity,^8^ consume fewer fruits and vegetables than people without stroke,^9^ and are more likely to be smokers than the general population.^10^ Nearly a third of stroke survivors do not adhere to their prescribed medications.^11^ Anxiety and depression affect between a quarter and a third of people who have experienced stroke respectively,^12^, ^13^ and many do not receive the psychological care they require.^14^, ^15^, ^16^ Alcohol consumption may follow a different pattern, with two studies finding that people who had experienced stroke were less likely to consume alcohol^9^ or engage in heavy alcohol consumption.^10^ The overall pattern of generally poor health risk factors after strokes suggests a need for interventions that improve the health of stroke survivors and reduce the risk of recurrent stroke.

There are also other factors which can affect or may be affected by a person's health risk factors. A person's physical function and independence in activities of daily living after stroke can negatively impact a person's ability to meet physical activity guidelines,^17^ and is associated with poststroke depression and anxiety.^18^, ^19^ People who live in rural and remote locations are more likely to engage in risky behaviours related to stroke such as smoking and drinking at harmful levels than people living in metropolitan areas.^20^ Relationships also exist between stroke health risk factors and other sociodemographic factors such as age, gender,^17^ income^21^ and Aboriginal and/or Torres Strait Islander status.^22^

Health risk factors are hard to change, and not everyone will experience the same combination of factors. In order to design health promotion campaigns and tailor interventions for secondary prevention of stroke, we need to better understand how these health risk factors interact and cluster with each other, and how they relate with other factors such as a person's sociodemographic status clinical characteristics, independence and physical function. Latent Class Analysis is a statistical technique designed to identify distinct subgroups or “clusters” of people who share common characteristics based on their responses to measured variables. This has previously been used to identify clusters of health risk behaviours in Australian populations^23^, ^24^ and older adults.^25^ This study will utilise the latent class analysis method as part of answering the following research questions:

1. What is the prevalence of health risk factors (physical activity levels, diet quality, alcohol consumption, smoking status, blood pressure medication use, and depression and anxiety levels) in a community‐dwelling sample of stroke survivors?
2. How do these health risk factors cluster, and are they related to physical functioning, independent living, or sociodemographic factors?

## 2 METHODS

### 2.1 Study design

This study is a secondary analysis of data obtained from the baseline survey of a national randomised controlled trial – Prevent 2nd Stroke.^26^ It was planned during the data collection phase of the main trial. The Prevent 2nd Stroke program is an online module‐based program aimed at improving the health‐related quality of life of stroke survivors through education and goal setting for stroke‐related health risk factors.^27^

All data presented here were collected as part of the trial.

### 2.2 Sample

Individuals were eligible to take part in the study if they were aged 18 years and over, 6‐36 months poststroke, sufficiently fluent in English and had access to the internet via a home device (eg computer, tablet, or smartphone) or willing to use public internet services (eg public library). Participants were required to be able to walk without assistance and have no more than a moderate disability, and so were ineligible if they scored ≤3 on the modified Rankin Scale.^28^

### 2.3 Procedures

The Prevent 2nd Stroke trial recruited participants from the Australian Stroke Clinical Registry that contributes data from select hospitals across New South Wales, Queensland, South Australia, Tasmania, Victoria, and the Australian Capital Territory based on an opt‐out process. This registry is designed to be a nationally representative sample of Australian Stroke Survivors. Additional participants were also recruited through the opt‐in Stroke Research Register, Hunter, which recruits stroke survivors from the Hunter region of NSW. Details regarding the study design and methods are published elsewhere.^26^ Ethical approval was obtained through the University of Newcastle Human Research Ethics Committee (H‐2017‐0051). Informed consent was obtained from all participants.

The baseline survey was administered via a computer‐assisted telephone interview and included questions about participant demographics and health risk factors. The full baseline survey can be viewed in the Supplementary Material. The sample used in this study consists of all participants who completed the baseline survey.

### 2.4 Measures

Seven health factor variables were measured:

1. *Diet quality*: The Australian Recommended Food Score (ARFS) questionnaire assessed diet quality and scores were categorised as “needs work” (<33), “getting there” (33‐38), “excellent” (39‐46), or “outstanding” (47+).^29^
2. *Physical activity*: The Godin‐Leisure‐Time Exercise Questionnaire assessed physical activity levels and categorised scores as “active” (24+), “moderately active” (14‐23) and “Sedentary” (0‐13).^30^
3. *Blood pressure medication*: Respondents were asked “Are you on blood pressure medications?” with response options “yes,” “no,” and “don't know.”
4. *Alcohol intake*: The Alcohol Use Disorders Identification Test – Consumption (AUDIT‐C) was used to assess alcohol intake. Respondents with a score of 3 or more in women or of 4 or more in men were considered to be drinking at risky levels.^31^
5. *Smoking status*: Respondents were asked, “do you currently smoke tobacco products?” with response options being “yes, daily,” “yes, once a week,” “yes, less than once a week” and “no.”^32^ Daily and occasional smokers were classified as current smokers.
6. *Anxiety*: Anxiety was measured using items 1 and 2 of the Patient Health Questionnaire‐4 (PHQ‐4).^33^ A score of three or more was considered positive for anxiety.
7. *Depression*: Depression was measured using items 3 and 4 of the PHQ‐4.^33^ A score of three or more was considered positive for depression.

The following explanatory variables were measured:

- *Demographic and clinical characteristics*: Age was classified (<55, 55‐64, 65‐74 and 74+ years); gender (male/female); weekly personal gross income was measured in Australian dollars and categorised into three groups of low (<$399), mid ($400‐999) and high ($1000+); country of birth (Australia/other); Aboriginal and/or Torres Strait Islander status; stroke type (stroke/transient ischaemic attack); and first stroke event (yes/no).
- *Remoteness Area*: Participant postcodes were matched with remoteness classifications from the Australian Bureau of Statistics,^34^ defining areas as Metropolitan (Major Cities) and NonMetropolitan (Inner Regional, Outer Regional, Remote, Very Remote).
- *Independent living and functioning*: The Barthel Index (BI)^35^, ^36^ was used to indicate participants' self‐care and mobility. Scores were assessed as “independent” (100), “slight dependence” (91‐99) and “moderate dependence” (61‐90). The Instrumental Activities of Daily Living (IADL) scale^37^ was used to indicate participants' ability to live independently in the community. A score of 8 indicated high function and independence, a score of 7 indicated relatively high function and independence, and a score of 6 or less indicated poorer function and independence.

### 2.5 Statistical analyses

Basic descriptive statistics (proportions and frequencies) were calculated for each of the variables.

A latent class analysis was performed with diet quality, physical activity, blood pressure, alcohol intake, smoking status, anxiety and depression variables. A latent class analysis was chosen Selection for the best number of latent classes to be produced was based on comparing Akaike information criterion (AIC) and Bayesian information criterion (BIC) measures of fit between models^38^; the model with the lowest BIC was chosen as the most appropriate. Corresponding response probabilities were determined for each item using this model and plotted accordingly.

To identify whether any of the participant characteristics were associated with resulting classes, separate univariate logistic regressions were performed. Aboriginal and Torres Strait Islander status was excluded from the logistic regression due to insufficient numbers. Crude odds ratios, 95% confidence intervals, number of observations used (N), pairwise *P*‐values and overall Type III *P*‐values were produced.

All statistical analyses were conducted using SAS v9.4 (SAS Institute, Cary, North Carolina, USA); *P* < .05 (two‐tailed) was used to indicate statistical significance.

## 3 RESULTS

There were 399 respondents to the baseline survey. Table 1 provides data on the participant characteristics of this sample. The majority were male (65%) and the average age was 66 years. More than half (71%) of the sample were at least moderately active and most (64%) had diet quality scores of “excellent” or “outstanding” and were living independently with high physical functioning.

**TABLE 1:** Sociodemographic and health risk factor characteristics (N = 399).

Characteristic	Measure	Mean (SD)
Age	Mean (SD)	66 (12)

Variable	Category	N (%)^a^
Sex	Male	260 (65)
Income (AUD)	Low <$399	109 (27)
	Mid $400‐$999	157 (39)
	High $1000+	96 (24)
	Do not know/Refused	34 (9)
Country of birth	Australia	308 (77)
Indigenous status	Aboriginal and/or Torres Strait Islander	3 (1)
Stroke type	Stroke	239 (60)
	Transient Ischaemic Attack (TIA)	142 (36)
	Do not know	18 (5)
First ever stroke	Yes	374 (94)
Remoteness	Metropolitan	230 (58)
	Nonmetropolitan	166 (42)
Independent living (IADL)	Independent (8)	326 (82)
	Slight dependence (7)	39 (10)
	Requires some assistance (0‐6)	31 (8)
Physical Functioning (Barthel Index)	Independent (100)	298 (75)
	Slight dependence (91‐99)	71 (18)
	Moderate dependence (61‐90)	24 (6)
Physical Activity (GLTEQ)	Sedentary (0‐13)	116 (29)
	Moderately active (14‐25)	101 (26)
	Active (24+)	179 (45)
Diet Quality (ARFS)	Needs Work (<33)	77 (19)
	Getting there (33‐38)	65 (16)
	Excellent (39‐46)	152 (38)
	Outstanding (47+)	101 (26)
Risky drinking (AUDIT‐C)	No	207 (52)
	Yes	189 (48)
Current Smoker	No	380 (96)
	Yes	16 (4)
Blood Pressure Medication	No	111 (28)
	Yes	275 (69)
	Do not know	10 (3)
Depression (PHQ‐4)	Low (0‐2)	347 (88)
	Depression (3‐6)	46 (12)
Anxiety (PHQ‐4)	Low (0‐2)	348 (88)
	Anxiety (3‐6)	46 (12)

Abbreviations: ARFS, Australian Recommended Food Score; AUD, Australian dollars; AUDIT‐C, Alcohol Use Disorder Identification Test – Consumption; GLTEQ, Godin‐Leisure‐Time Exercise Questionnaire; IADL, Instrumental Activities of Daily Living; PHQ‐4, Patient Health Questionnaire – 4.

^a^ Not all variable data add to 100% due to missing data.

Latent classes of between 2 and 10 classes were explored. Model selection identified two latent classes to be the most optimal (BIC 354.12, AIC 270.51). Table 2 provides the item response probabilities for each class. Based on their health risk factors, 16% of participants in this study fell into Class 1, and 84% fell into Class 2. Class 1 is characterised by individuals with a higher probability of lower diet quality (55% probability of “needing work” or “getting there”), lower physical activity (53% probability of being sedentary), higher levels of anxiety (59% probability) and depression (55% probability), but a lower probability of risky drinking (36% probability). For this paper Class 1 has been labelled “*Low Mood, Food & Moves Risk*.” Members of class 2 had a higher probability of a better quality diet (69% probability of having an “excellent” or “outstanding” diet), greater physical activity (51% probability of being “active”) but heavier drinking (51% probability) as well as lower probabilities of having anxiety (0.1% probability) or depression (1% probability). Class 2 has been labelled “*Alcohol Use Risk*.” Neither class was well‐differentiated by blood pressure medication usage or current smoking with similar probabilities produced for both groups.

**TABLE 2:** Latent class analysis: Probability of having each health risk factor for each class.

Health risk behaviour	Level	Class/Group^a^	
		Low mood, food and moves risk (16%)	Alcohol use risk (84%)
Physical Activity (GLTEQ)	Sedentary (0‐13)	**0.525**	0.263
	Moderately active (14‐25)	0.247	0.257
	Active (24+)	0.288	**0.507**
Diet Quality (ARFS)	Needs Work (<33)	0.357	0.155
	Getting there (33‐38)	0.189	0.159
	Excellent (39‐46)	0.329	0.399
	Outstanding (47+)	0.125	0.288
Risky drinking (AUDIT‐C)	No	**0.645**	0.493
	Yes	0.355	**0.507**
Current smoker	No	**0.888**	**0.977**
	Yes	0.112	0.023
Blood Pressure Medication	No	0.264	0.293
	Yes	**0.736**	**0.707**
Depression (PHQ‐4)	Low (0‐2)	0.455	**0.986**
	Depression (3‐6)	**0.545**	0.014
Anxiety (PHQ‐4)	Low (0‐2)	0.410	**0.999**
	Anxiety (3‐6)	**0.590**	0.001

Abbreviations: ARFS, Australian Recommended Food Score; AUDIT‐C, Alcohol Use Disorder Identification Test – Consumption; GLTEQ, Godin‐Leisure‐Time Exercise Questionnaire; PHQ‐4, Patient Health Questionnaire – 4.

^a^ Bolded cells correspond to the majority category of health risk behaviour (>50%) for each class.

Table 3 displays associations between socio‐demographic or clinical characteristics and latent class group membership (class 1 vs class 2, where class 2 is the reference level). Only physical functioning (*P* = .0002) and independent living (*P* < .0001) variables were found to be significantly associated with latent class allocation, based on respective crude *P*‐values. Participants also had higher odds of being in the *Low Mood, Food and Moves Risk* group if their Barthel Index score indicated moderate (OR: 5.969; 95% CI: 2.492‐14.300) or slight dependence in ADLs (OR: 1.889; 95% CI: 0.971‐3.677) or with IADL scores of 0‐6 (OR: 5.020; 95% CI: 2.290‐11.006) or 7 (OR: 2.397; 95% CI: 1.088‐5.280). From Type III tests, no associations between other characteristics and latent class membership were significant.

**TABLE 3:** Estimates of individual crude logistic regression models for latent class allocation (modelling probability of being in the low mood, food and moves risk group).

Model	N		Odds ratio (95% CI)	Pairwise *P*‐value	Type III *P*‐value
Age (years)	395	55‐64 vs <55	0.94 (0.38‐2.31)	.65	.44
		65‐74 vs <55	0.94 (0.42‐2.11)	.57	
		74+ vs <55	0.55 (0.23‐1.36)	.10	
Gender	396	Female vs Male	0.98 (0.56‐1.71)	.93	.93
Income	362	High ($1000+) vs Low (<$399)	0.46 (0.19‐1.11)	.03	.06
		Mid ($400‐$999) vs Low (<$399)	1.24 (0.66‐2.36)	.04	
Country of birth	395	Other vs Australia	0.84 (0.44‐1.63)	.61	.61
Stroke type	378	Stroke vs Transient Ischaemic Attack	1.21 (0.68‐2.15)	.51	.51
More than one stroke	396	No vs Yes	1.23 (0.35‐4.30)	.74	.74
Remoteness area	393	Metropolitan vs NonMetropolitan	1.25 (0.72‐2.17)	.43	.43
Physical functioning (BI)	393	Moderate Dependence (61‐90) vs Independent (100)	5.97 (2.49‐14.30)	<.01	<.01
		Slight Dependence (91‐99) vs Independence (100)	1.89 (0.97‐3.68)	.48	
Independent living (IADL)	396	0‐6 vs 8	5.02 (2.29‐11.01)	.01	<.01
		7 vs 8	2.40 (1.09‐5.28)	.87	

Abbreviations: BI, Barthel Index; IADL, Instrumental Activities of Daily Living.

## 4 DISCUSSION

In investigating the prevalence of health risk factors in this sample of stroke survivors, we found 29% were insufficiently active, 35% did not have diet scores in line with Australian dietary guidelines, 48% were drinking at risky levels, 4% were smoking, 28% were not on any blood pressure medication, 12% scored positively for anxiety and 12% scored positively for depression. Two classes of health risk factors were identified during the latent class analysis – *Low Mood, Food & Moves Risk* and *Alcohol Use Risk*. One in six participants were in the *Low Mood, Food & Moves Risk* group who generally had lower levels of physical activity, poorer diet quality and higher levels of depression and anxiety, but a lower probability of risky drinking. Lower levels of independence and physical functioning were predictor variables for this group. In contrast, the participants in the *Alcohol Use Risk* group had better physical activity and diet quality scores, significantly lower probability of depression and anxiety, but a higher probability of risky drinking.

We found that the prevalence of stroke‐related health risk factors was lower in our sample than what has previously been found in other stroke survivor population samples. Compared to our findings, studies among people who have experienced stroke have found higher rates of insufficient physical activity (56.5%),^10^ low fruit and vegetable uptake (51.7%),^10^ and higher rates of depression (31%)^12^ and anxiety (24%).^13^ It should be noted that the measures used to assess health risk factors across studies varies making direct comparison between studies difficult. Our sample consisted of participants in a randomised controlled trial who had to meet a modified Rankin Scale of ≤3 to be eligible which indicates that no participants had more than moderate disability and all were able to walk without assistance. Additionally, it is possible that stroke survivors already conscious of their health risk factors were more drawn to participate in a trial that addressed these factors.

One in six of our relatively “well” and “healthy” sample were in the *Low Mood, Food & Moves Risk* group and had a higher probability of engaging in multiple risk factors relating to poor diet quality, low physical activity, depression and anxiety. These participants had higher odds of being part of this group if they had lower Instrumental Activities of Daily Living and Barthel index scores indicating lower levels of independence in everyday living. Previous studies have also identified that people with higher levels of poststroke disability have more difficulty engaging in activities to improve their health risk behaviours due to physical limitations^39^, ^40^ and that poststroke depression can increase functional impairment and disability.^41^, ^42^, ^43^ The effects of poststroke disability need to be taken into account when designing programs targeting these risk factors in people who have had stroke. Programs originally designed for the general population may be adapted for use by people who have experienced strokes though changes such as simplification of the visual presentation of the program including use of pictures and reduced wordiness, focus on daily physical activity rather than activities that might not be accessible to all people with stroke, optional involvement of a care‐partner, and inclusion of stroke‐specific information.^44^ Co‐design of poststroke programs with people who have experienced stroke should be considered to ensure programs are accessible, relevant and interesting to people with stroke.^45^, ^46^ Future research would benefit from assessing how health risk factors cluster in people with moderate to severe poststroke disability. This would aid in further tailoring interventions that can target risk factors relevant to different levels of disability and design them to take the particular needs of people with stroke into account.

Risky levels of alcohol consumption after stroke may be an overlooked problem that requires further investigation. Members of the *Alcohol Use Risk* group, who were more likely to meet guidelines for other health risk factors, had a more than 50% probability of drinking at risky levels according to the AUDIT‐C, and 48% of the total sample were participating in risky drinking activities, which was a surprising finding. This requires further understanding given a large recent study shows that previously accepted protective effects of moderate alcohol intake are largely noncausal and alcohol consumption uniformly increases stroke risk and blood pressure.^47^ A previous study found that only 5.4% of people with stroke participated in heavy drinking, though this was measured using a different tool to our study and is not directly comparable.^10^ While a clear understanding of the drinking patterns of the broader population of people with stroke is lacking, this pattern may not be unusual with risky alcohol consumption among older Australians increasing over the last decade.^48^ Chapman et al^49^ analysed the drinking habits of Australians based on the National Drug Strategy Household Survey data and identified that 16.8% of people aged over 50 reported drinking at levels associated with long term risk and 17% reported drinking levels which placed them at short term risk. Less than half of these risky drinkers perceived their drinking as harmful. There is a need to better understand patterns of alcohol consumption in adults with stroke, as well as their reasons for consumption and awareness of the associated risks. This will be important to both design and deliver appropriate interventions to reduce their alcohol intake and risk of recurrent stroke, as well as to understand if current guidelines require further clarity to avoid mixed messaging.

### 4.1 Study limitations and strengths

This study provides data from a large, national sample of stroke survivors. The eligibility criteria required that participants had a modified Rankin Score of ≤3 which resulted in a sample with high levels of independence and limited disability. This means it cannot be generalised to the broader stroke population due to the exclusion of people who experience greater levels of disability after stroke. Furthermore, the main study outcomes of health risk factors are dependent on self‐report measures and may be underestimations.

## 5 CONCLUSION

This study provides insight into the health risk behaviour profiles of stroke survivors living in the community. Two groups were identified. One group had a higher likelihood of lower levels of physical activity, poorer diet quality and higher levels of depression and anxiety. Participants were more likely to be in this group if they had low levels of independence and physical functioning. The other group had no identified predictor variables and participants in this group were more likely to meet guidelines for diet and exercise with a low probability of depression or anxiety, but had a >50% probability of drinking at risky levels. It highlights how, even in stroke survivors with relatively fewer effects of disability and in general good health, continued support is required to improve health risk behaviours to reduce risk of recurrent stroke. Future interventions should be tailored to and target the needs and requirements of the relevant health behaviour profile. Risky levels of alcohol consumption may also be an overlooked problem for this population that requires further investigation to understand the causes and breadth of the problem.

## FUNDING INFORMATION

This work was supported by a grant from the National Health and Medical Research Council (APP1125429). The University of Newcastle provided funding for statistical support and a Research Training Program scholarship for Brigid Clancy. Ashleigh Guillaumier was supported by a postdoctoral fellowship from the Heart Foundation. Amanda L. Baker was supported by a NHMRC Research Fellowship. No funders had a role in study design, data collection, data analysis, data interpretation, or writing or publication of the report.

## CONFLICT OF INTEREST STATEMENT

The authors declare no conflict of interest.

## ETHICS STATEMENT

Ethical approval was obtained through the University of Newcastle Human Research Ethics Committee (H‐2017‐0051). Informed consent was obtained from all participants.

## Supporting information


**Data S1.** Supporting Information

## Data Availability

The data that support the findings of this study are available from the corresponding author upon reasonable request.

## References

[1] GBD 2016 Lifetime Risk of Stroke Collaborators . Global, regional, and country‐specific lifetime risks of stroke, 1990 and 2016. N Engl J Med. 2018;379(25):2429–37. 30575491 10.1056/NEJMoa1804492 PMC6247346

[2] Johnson CO , Nguyen M , Roth GA , Nichols E , Alam T , Abate D , et al. Global, regional, and national burden of stroke, 1990–2016: a systematic analysis for the Global Burden of Disease Study 2016. Lancet Neurol. 2019;18(5):439–58. 30871944 10.1016/S1474-4422(19)30034-1 PMC6494974

[3] Mohan KM , Wolfe CDA , Rudd AG , Heuschmann PU , Kolominsky‐Rabas PL , Grieve AP . Risk and cumulative risk of stroke recurrence. Stroke. 2011;42(5):1489–94. 21454819 10.1161/STROKEAHA.110.602615

[4] O'Donnell MJ , Chin SL , Rangarajan S , Xavier D , Liu L , Zhang H , et al. Global and regional effects of potentially modifiable risk factors associated with acute stroke in 32 countries (INTERSTROKE): a case‐control study. Lancet. 2016;388(10046):761–75. 27431356 10.1016/S0140-6736(16)30506-2

[5] Wu Q‐E , Zhou A‐M , Han Y‐P , Liu Y‐M , Yang Y , Wang X‐M , et al. Poststroke depression and risk of recurrent stroke. Medicine (Baltimore). 2019;98(42):e17235. 31626084 10.1097/MD.0000000000017235 PMC6824697

[6] Pérez‐Piñar M , Ayerbe L , González E , Mathur R , Foguet‐Boreu Q , Ayis S . Anxiety disorders and risk of stroke: a systematic review and meta‐analysis. Eur Psychiatry. 2017;41(1):102–8. 28135591 10.1016/j.eurpsy.2016.11.004

[7] Samsa GP , Bian J , Lipscomb J , Matchar DB . Epidemiology of recurrent cerebral infarction. Stroke. 1999;30(2):338–49. 9933269 10.1161/01.str.30.2.338

[8] Fini NA , Holland AE , Keating J , Simek J , Bernhardt J . How physically active are people following stroke? Systematic review and quantitative synthesis. Phys Ther. 2017;97(7):707–17. 28444348 10.1093/ptj/pzx038

[9] Wali H , Kurdi S , Bilal J , Riaz IB , Bhattacharjee S . Health behaviors among stroke survivors in the United States: a propensity score‐matched study. J Stroke Cerebrovasc Dis. 2018;27(8):2124–33. 29673613 10.1016/j.jstrokecerebrovasdis.2018.03.013

[10] Bailey RR , Phad A , McGrath R , Haire‐Joshu D . Prevalence of five lifestyle risk factors among U.S. adults with and without stroke. Disabil Health J. 2019;12(2):323–7. 30448248 10.1016/j.dhjo.2018.11.003 PMC6431268

[11] Al Alshaikh S , Quinn T , Dunn W , Walters M , Dawson J . Predictive factors of non‐adherence to secondary preventative medication after stroke or transient ischaemic attack: a systematic review and meta‐analyses. Eur Stroke J. 2016;1(2):65–75. 29900404 10.1177/2396987316647187 PMC5992740

[12] Hackett ML , Pickles K . Part I: frequency of depression after stroke: an updated systematic review and meta‐analysis of observational studies. Int J Stroke. 2014;9(8):1017–25. 25117911 10.1111/ijs.12357

[13] Knapp P , Dunn‐Roberts A , Sahib N , Cook L , Astin F , Kontou E , et al. Frequency of anxiety after stroke: an updated systematic review and meta‐analysis of observational studies. Int J Stroke. 2020;15(3):244–55. 31980004 10.1177/1747493019896958

[14] Stroke Foundation . National stroke audit – rehabilitation services report. Melbourne, Australia: Stroke Foundation; 2020.

[15] Klinedinst NJ , Dunbar SB , Clark PC . Stroke survivor and informal caregiver perceptions of poststroke depressive symptoms. J Neurosci Nurs. 2012;44(2):72–81. 22367269 10.1097/JNN.0b013e3182477944 PMC3296963

[16] Calleo J , Amspoker AB , Marsh L , Kunik ME . Mental health diagnoses and health care utilization in persons with dementia, Parkinson's disease, and stroke. J Neuropsychiatry Clin Neurosci. 2015;27(2):e117–e21. 25541867 10.1176/appi.neuropsych.13110333

[17] Thilarajah S , Mentiplay BF , Bower KJ , Tan D , Pua YH , Williams G , et al. Factors associated with post‐stroke physical activity: a systematic review and meta‐analysis. Arch Phys Med Rehabil. 2018;99(9):1876–89. 29056502 10.1016/j.apmr.2017.09.117

[18] Ghaffari A , Rostami HR , Akbarfahimi M . Predictors of instrumental activities of daily living performance in patients with stroke. Occup Ther Int. 2021;2021:6675680–7. 33727902 10.1155/2021/6675680 PMC7936883

[19] Lee EH , Kim JW , Kang HJ , Kim SW , Kim JT , Park MS , et al. Association between anxiety and functional outcomes in patients with stroke: a 1‐year longitudinal study. Psychiatry Investig. 2019;16(12):919–25. 10.30773/pi.2019.0188 PMC6933133 31698556

[20] Australian Institute of Health and Welfare . Rural and remote health. Canberra: AIHW; 2022.

[21] Australian Institute of Health and Welfare . Health across socioeconomic groups. Canberra: AIHW; 2022.

[22] Australian Institute of Health and Welfare . Determinants of health for indigenous Australians. Canberra: AIHW; 2022.

[23] Noble NE , Paul CL , Turner N , Blunden SV , Oldmeadow C , Turon HE . A cross‐sectional survey and latent class analysis of the prevalence and clustering of health risk factors among people attending an Aboriginal Community Controlled Health Service. BMC Public Health. 2015;15(1):666. 26173908 10.1186/s12889-015-2015-8 PMC4502927

[24] Hutchesson MJ , Duncan MJ , Oftedal S , Ashton LM , Oldmeadow C , Kay‐Lambkin F , et al. Latent class analysis of multiple health risk behaviors among Australian university students and associations with psychological distress. Nutrients. 2021;13(2):425. 33525585 10.3390/nu13020425 PMC7912169

[25] Liao J , Mawditt C , Scholes S , Lu W , Umeda M , Muniz Terrera G , et al. Similarities and differences in health‐related behavior clustering among older adults in eastern and Western countries: a latent class analysis of global aging cohorts. Geriatr Gerontol Int. 2019;19(9):930–7. 31309695 10.1111/ggi.13737

[26] Guillaumier A , McCrabb S , Spratt NJ , Pollack M , Baker AL , Magin P , et al. An online intervention for improving stroke survivors' health‐related quality of life: study protocol for a randomised controlled trial. Trials. 2019;20(1):491. 31399140 10.1186/s13063-019-3604-0 PMC6688335

[27] Denham AMJ , Guillaumier A , McCrabb S , Turner A , Baker A , Spratt N , et al. Development of an online secondary prevention programme for stroke survivors: prevent 2nd stroke. BMJ Innov. 2019;5:35–42.

[28] Quinn TJ , Lees KR , Hardemark H‐G , Dawson J , Walters MR . Initial experience of a digital training resource for modified Rankin scale assessment in clinical trials. Stroke. 2007;38(8):2257–61. 17600236 10.1161/STROKEAHA.106.480723

[29] Collins CE , Burrows TL , Rollo ME , Boggess MM , Watson JF , Guest M , et al. The comparative validity and reproducibility of a diet quality index for adults: the Australian Recommended Food Score. Nutrients. 2015;7(2):785–98. 25625814 10.3390/nu7020785 PMC4344560

[30] Eisenmann J , Milburn N , Jacobsen L , Moore S . Reliability and convergent validity of the Godin Leisure‐Time Exercise Questionnaire in rural 5th‐grade school‐children. J Hum Mov Stud. 2002;43(2):135–49.

[31] Bush K , Kivlahan DR , McDonell MB , Fihn SD , Bradley KA . The AUDIT alcohol consumption questions (AUDIT‐C): an effective brief screening test for problem drinking. Arch Intern Med. 1998;158(16):1789–95. 9738608 10.1001/archinte.158.16.1789

[32] Mullins R , Borland R . Changing the way smoking is measured among Australian adults: a preliminary investigation of Victorian data. Quit Eval Stud. 1998;9:163–73.

[33] Kroenke K , Spitzer RL , Williams JB , Löwe B . An ultra‐brief screening scale for anxiety and depression: the PHQ–4. Psychosomatics. 2009;50(6):613–21. 19996233 10.1176/appi.psy.50.6.613

[34] Australian Bureau of Statistics . 1270.0.55.005 ‐ Australian Statistical Geography Standard (ASGS): Volume 5 ‐ Remoteness Structure, July 2016 [updated 16/03/2018]. Available from: https://www.abs.gov.au/AUSSTATS/abs@.nsf/DetailsPage/1270.0.55.005July%202016?OpenDocument.

[35] Middleton S , Levi C , Ward J , Grimshaw J , Griffiths R , D'Este C , et al. Fever, hyperglycaemia and swallowing dysfunction management in acute stroke: a cluster randomised controlled trial of knowledge transfer. Implement Sci. 2009;4(1):16. 19291323 10.1186/1748-5908-4-16 PMC2663544

[36] Mahoney FI , Barthel DW . Functional evaluation: The Barthel Index: A simple index of independence useful in scoring improvement in the rehabilitation of the chronically ill. Md State Med J. 1965;14:61–5. 14258950

[37] Katz S . Assessing self‐maintenance: activities of daily living, mobility, and instrumental activities of daily living. J Am Geriatr Soc. 1983;31(12):721–7. 6418786 10.1111/j.1532-5415.1983.tb03391.x

[38] Lanza ST , Rhoades BL . Latent class analysis: an alternative perspective on subgroup analysis in prevention and treatment. Prev Sci. 2013;14(2):157–68. 21318625 10.1007/s11121-011-0201-1 PMC3173585

[39] Field MJ , Gebruers N , Shanmuga Sundaram T , Nicholson S , Mead G . Physical activity after stroke: a systematic review and meta‐analysis. ISRN Stroke. 2013;2013:1–13.

[40] Nicholson S , Sniehotta FF , Van Wijck F , Greig CA , Johnston M , McMurdo MET , et al. A systematic review of perceived barriers and motivators to physical activity after stroke. Int J Stroke. 2013;8(5):357–64. 22974010 10.1111/j.1747-4949.2012.00880.x

[41] Paolucci S , Iosa M , Coiro P , Venturiero V , Savo A , De Angelis D , et al. Post‐stroke depression increases disability more than 15% in ischemic stroke survivors: a case‐control study. Front Neurol. 2019;10:926. 31507525 10.3389/fneur.2019.00926 PMC6718567

[42] Astuti P , Kusnanto K , Dwi NF . Depression and functional disability in stroke patients. J Public Health Res. 2020;9(2):1835. 32728574 10.4081/jphr.2020.1835 PMC7376455

[43] Schöttke H , Gerke L , Düsing R , Möllmann A . Post‐stroke depression and functional impairments – a 3‐year prospective study. Compr Psychiatry. 2020;99:152171. 32179262 10.1016/j.comppsych.2020.152171

[44] Bailey RR , Stevenson JL , Driver S , McShan E . Health behavior change following stroke: recommendations for adapting the diabetes prevention program‐group lifestyle balance program. Am J Lifestyle Med. 2022;16(2):221–8. 35370513 10.1177/1559827619897252 PMC8971701

[45] Ramage ER , Burke M , Galloway M , Graham ID , Janssen H , Marsden DL , et al. Fit for purpose. Co‐production of complex behavioural interventions. A practical guide and exemplar of co‐producing a telehealth‐delivered exercise intervention for people with stroke. Health Res Policy Syst. 2022;20(1):2. 34980156 10.1186/s12961-021-00790-2 PMC8722305

[46] Zacharia K , Patterson AJ , English C , Ramage E , Galloway M , Burke M , et al. i‐rebound after stroke‐eat for health: Mediterranean dietary intervention co‐design using an integrated knowledge translation approach and the TIDieR checklist. Nutrients. 2021;13(4):1058. 33805076 10.3390/nu13041058 PMC8064089

[47] Millwood IY , Walters RG , Mei XW , Guo Y , Yang L , Bian Z , et al. Conventional and genetic evidence on alcohol and vascular disease aetiology: a prospective study of 500 000 men and women in China. Lancet. 2019;393(10183):1831–42. 30955975 10.1016/S0140-6736(18)31772-0 PMC6497989

[48] Roche AM , Kostadinov V . Baby boomers and booze: we should be worried about how older Australians are drinking. Med J Aust. 2019;210(1):38–9. 30636302 10.5694/mja2.12025

[49] Chapman J , Harrison N , Kostadinov V , Skinner N , Roche A . Older Australians' perceptions of alcohol‐related harms and low‐risk alcohol guidelines. Drug Alcohol Rev. 2020;39(1):44–54. 31829473 10.1111/dar.13022

