# Supplementary material for: Health risk factors in Australian Stroke Survivors: A latent class analysis
Source: Health Promot J Austr. 2023 Feb 28;35(1):37–44. doi: 10.1002/hpja.706 (PMC10952979; doi:10.1002/hpja.706)
Supplement: Supplementary file 1 — Data S1. Supporting Information [file HPJA-35-37-s001.docx]

Participant ID

**
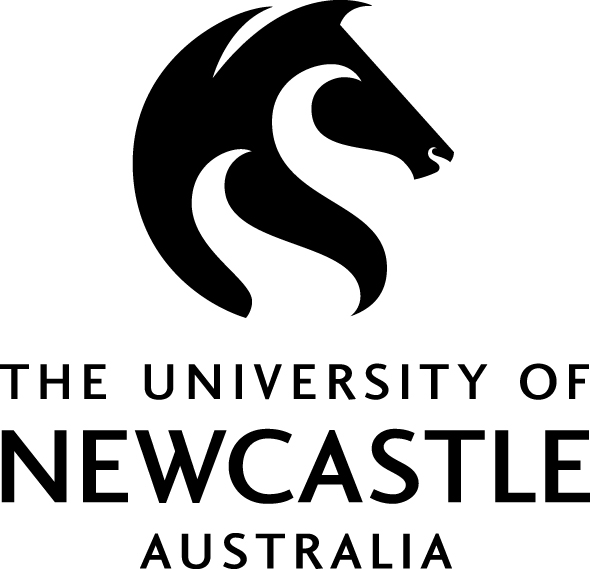
**

**The “Prevent 2^nd^ Stroke” Study**

Participant Survey

Thank you for agreeing to complete our survey.

This survey will ask you a number of questions about your health.

It should take you about 30 minutes to complete.

***Demographics***

**1) Which state do you live in?**

| New South Wales | 1 |
| --- | --- |
| Queensland | 2 |
| South Australia | 3 |
| Tasmania | 4 |
| Victoria | 5 |
| Western Australia | 6 |

**2) What is your post code?**

**3) In what year were you born?**

**4) What gender do you identify with?**

| Male | 1 |
| --- | --- |
| Female | 2 |
| Non-binary | 3 |
| Other (Please specify) | 4 |
| *Don’t know (don’t read out)* | *5* |
| *Refused (don’t read out)* | *R* |

***Stroke event***

*To start with, I’d like to confirm some details about your stroke or TIA event.*

**5) How long ago was your stroke or Transient Ischaemic Attack (TIA)?**

**Months** (max 11) **or**

**Years** (max 20)

**6) Have you experienced a stroke or TIA prior to this episode?**

| No | 1 | Go to Q7 |
| --- | --- | --- |
| Yes, had stroke before | 2 | End survey (ineligible) |
| Yes, had TIA before | 3 |  |
| Yes, had both stroke and a TIA before | 4 |  |
| *Don’t know (don’t read out)* | *5* | Go to Q7 |
| *Refused (don’t read out)* | *R* | Go to Q7 |

***Health-related quality of life***

**7) Under each heading, please identify the option that best describes your health TODAY**

**a. MOBILITY**

| I have no problems with walking around | 1 |
| --- | --- |
| I have slight problems with walking around | 2 |
| I have moderate problems with walking around | 3 |
| I have severe problems with walking around | 4 |
| I am unable to walk around | 5 |
| *Don’t know (don’t read out)* | *6* |
| *Refused (don’t read out)* | *R* |

**b. PERSONAL CARE**

| I have no problems with washing or dressing myself | 1 |
| --- | --- |
| I have slight problems with washing or dressing myself | 2 |
| I have moderate problems with washing or dressing myself | 3 |
| I have severe problems with washing or dressing myself | 4 |
| I am unable to wash or dress myself | 5 |
| *Don’t know (don’t read out)* | *6* |
| *Refused (don’t read out)* | *R* |

**c. USUAL ACTIVITIES** (e.g. work, study, housework, family or leisure activities)

| I have no problems doing my usual activities | 1 |
| --- | --- |
| I have slight problems doing my usual activities | 2 |
| I have moderate problems doing my usual activities | 3 |
| I have severe problems doing my usual activities | 4 |
| I am unable to do my usual activities | 5 |
| *Don’t know (don’t read out)* | *6* |
| *Refused (don’t read out)* | *R* |

**d. PAIN/DISCOMFORT**

| I have no pain or discomfort | 1 |
| --- | --- |
| I have slight pain or discomfort | 2 |
| I have moderate pain or discomfort | 3 |
| I have severe pain or discomfort | 4 |
| I have extreme pain or discomfort | 5 |
| *Don’t know (don’t read out)* | *6* |
| *Refused (don’t read out)* | *R* |

**e. ANXIETY/DEPRESSION**

| I am not anxious or depressed | 1 |
| --- | --- |
| I am slightly anxious or depressed | 2 |
| I am moderately anxious or depressed | 3 |
| I am severely anxious or depressed | 4 |
| I am extremely anxious or depressed | 5 |
| *Don’t know (don’t read out)* | *6* |
| *Refused (don’t read out)* | *R* |

**8)** **We would like to know how good or bad your health is TODAY.**

Imagine a scale numbered from 0 to 100, where 100 means the best health you can imagine and 0 means the worst health you can imagine.

We would like to know how good or bad your health is TODAY?

What number would you say your health is today 0 – 100 remember 100 means best health.

*Code ‘888’ if don’t know*

*Code ‘999’ if refused*

***Smoking***

**9) Do you currently smoke tobacco products?**

| Yes, daily | 1 |
| --- | --- |
| Yes, at least once a week | 2 |
| Yes, but less often than once a week | 3 |
| Not at all | 4 |
| *Don’t know (don’t read out)* | *5* |
| *Refused (don’t read out)* | *R* |

**10) Have you smoked at least 100 cigarettes or a similar amount of tobacco in your life?**

| Yes | 1 |
| --- | --- |
| No | 2 |
| Not sure | 3 |
| *Refused (don’t read out)* | *R* |

***Alcohol***

**11) Have you had an alcoholic drink of any kind in the last 12 months?**

| Yes | 1 | Go to Q12 |
| --- | --- | --- |
| No | 2 | Go to Q15 |
| *Don’t know (don’t read out)* | *3* | Go to Q15 |
| *Refused (don’t read out)* | *R* | Go to Q15 |

**12) How often did you have a drink containing alcohol in the past year?**

| Never | 1 | Go to Q15 |
| --- | --- | --- |
| Monthly or less | 2 | Go to Q13 |
| 2 to 4 times a month | 3 | Go to Q13 |
| 2 to 3 times a week | 4 | Go to Q13 |
| 4 or more times a week | 5 | Go to Q13 |
| *Don’t know (don’t read out)* | *3* | Go to Q13 |
| *Refused (don’t read out)* | *R* | Go to Q13 |

***Please use the definitions of Standard Drinks provided below to assist participant to answer the following questions.***


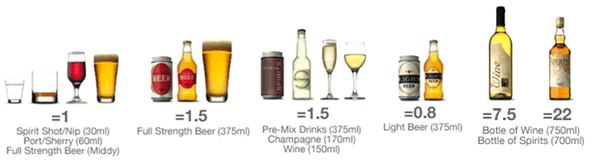


**13) How many standard drinks containing alcohol did you have on a typical day when you were drinking in the past year?**

| 1 to 2 drinks | 1 |
| --- | --- |
| 3 to 4 drinks | 2 |
| 5 to 6 drinks | 3 |
| 7 to 9 drinks | 4 |
| 10 or more drinks | 5 |
| *Don’t know (don’t read out)* | *6* |
| *Refused (don’t read out)* | *R* |

**14) How OFTEN do you have four or more standard drinks on one occasion in the past year?**

| Never | 1 |
| --- | --- |
| Less than monthly | 2 |
| Monthly | 3 |
| Weekly | 4 |
| Daily or almost daily | 5 |
| *Don’t know* | *6* |
| *Refused* | *R* |

***Blood pressure***

**15) Do you know your blood pressure?**

| Yes | 1 | Go to Q55 |
| --- | --- | --- |
| No | 2 | Go to Q56 |
| *Don’t know* | *3* | Go to Q56 |
| *Refused* | *R* | Go to Q56 |

**16) What is your blood pressure?**

**Top number: ­­­­­­­­­­­­___________**

**Bottom number: ________**

**17) Are you on medication for high blood pressure?**

| Yes | 1 |
| --- | --- |
| No | 2 |
| *Don’t know* | *3* |
| *Refused* | *R* |

***Depression and Anxiety***

**18) Over the last 2 weeks, how often have you been bothered by any of the following problems?**

|  | **Not at all** | **Several days** | **More than half the days** | **Nearly every day** | ***Don’t know***  *(don’t read out)* | ***Refused***  *(don’t read out)* |
| --- | --- | --- | --- | --- | --- | --- |
| Little interest or pleasure in doing things. | 1 | 2 | 3 | 4 | *5* | *R* |
| Feeling down, depressed, or hopeless. | 1 | 2 | 3 | 4 | *5* | *R* |
| Feeling nervous, anxious, or on edge | 1 | 2 | 3 | 4 | *5* | *R* |
| Not being able to stop or control worrying | 1 | 2 | 3 | 4 | *5* | *R* |

***Physical functioning and independent living***

*****Barthel index (replaces Activities of Daily Living to measure physical functioning)*****

**19) The next questions ask you for some specific details about your activities of daily living. Please tell me how well you can carry out the following tasks.**

1. **How able are you to feed yourself**

| Unable | 1 |
| --- | --- |
| Needs help cutting, spreading butter, etc., or requires modified diet | 2 |
| Independent | 3 |
| *Refused (don’t read out)* | *R* |

1. **How able are you to bath yourself**

| Dependent (0) | 1 |
| --- | --- |
| Independent (or in shower) (5) | 2 |
| *Refused (don’t read out)* | *R* |

1. **How able are you to take care of your grooming**

| Dependent (0) | 1 |
| --- | --- |
| Needs help with personal care | 2 |
| Independent face / hair / teeth / shaving (implements provided) (5) | 3 |
| *Refused (don’t read out)* | *R* |

1. **How able are you to dress yourself**

| Dependent (0) | 1 |
| --- | --- |
| Needs help but can do about half unaided (5) | 2 |
| Independent (including button, zips and laces etc) (10) | 3 |
| *Refused (don’t read out)* | *R* |

1. **How able are you to manage bowel movements yourself**

| Incontinent (or needs to be given enemas) (0) | 1 |
| --- | --- |
| Occasional accident (5) | 2 |
| Continent (10) | 3 |
| *Refused (don’t read out)* | *R* |

1. **How able are you to bladder control yourself**

| Incontinent or catheterized and unable to manage alone (0) | 1 |
| --- | --- |
| Occasional accident (5) | 2 |
| Continent (10) | 3 |
| *Refused (don’t read out)* | *R* |

1. **How able are you to use the toilet independently**

| Dependent (0) | 1 |
| --- | --- |
| Needs help but can do something alone (5) | 2 |
| Independent (on and off, dressing and wiping) (10) | 3 |
| *Refused (don’t read out)* | *R* |

1. **How able are you to transfer from bed to chair and back yourself**

| Unable, no sitting balance (0) | 1 |
| --- | --- |
| Major help (one or two people, physical) can sit (5) | 2 |
| Minor help (verbal or physical) (10) | 3 |
| Independent (15) |  |
| *Refused (don’t read out)* | *R* |

1. **How mobile are you**

| Immobile or < 50 metres | 1 |
| --- | --- |
| Wheelchair independent, including corners, > 50 metres | 2 |
| Walks with help of one person (verbal or physical) > 50 metres | 3 |
| Independent (but may use any aid; for example, stick) > 50 metres | 4 |
| *Refused (don’t read out)* | *R* |

1. **How able are you to use stairs**

| Unable | 1 |
| --- | --- |
| Needs help (verbal, physical, carrying aid) | 2 |
| Independent | 3 |
| *Refused (don’t read out)* | *R* |

*****Updated wording of Instrumental Activities of Daily Living (IADL) for CATI*****

***20) We are now going to ask you some other questions around activities of daily living using a different scale for your answers.***

1. **Do you use the telephone?**

| No, does not use telephone at all | 1 |
| --- | --- |
| Yes, answers telephone but does not dial | 2 |
| Yes, dials a few well-known numbers | 3 |
| Yes, operates telephone on own initiative-looks up and dials numbers, etc. | 4 |
| *Don’t Know (Don’t Read out)* | *5* |
| *Refused (Don’t Read out)* | *R* |

1. **Do you do the shopping?**

| No, completely unable to shop | 1 |
| --- | --- |
| Yes, needs to be accompanied on any shopping trip | 2 |
| Yes, shops independently for small purchases | 3 |
| Yes, takes care of all shopping needs independently | 4 |
| *Don’t Know (Don’t Read out)* | *5* |
| *Refused (Don’t Read out)* | *R* |

1. **Do you do any food preparation?**

| No, needs to have meals prepared and served | 1 |
| --- | --- |
| Yes, heats, serves and prepares meals, or prepares meals, or prepares meals but does not maintain adequate diet | 2 |
| Yes, prepares adequate meals if supplied with ingredients | 3 |
| Yes, plans, prepares and serves adequate meals  independently | 4 |
| *Don’t Know (Don’t Read out)* | *5* |
| *Refused (Don’t Read out)* | *R* |

1. **Do you do any housework?**

| No, does not participate in any house work | 1 |
| --- | --- |
| No, needs help with all home maintenance tasks | 2 |
| Yes, performs light daily tasks but cannot maintain an acceptable level of cleanliness. | 3 |
| Yes, performs light daily tasks such as dish washing, bed making | 4 |
| Yes, maintains the house alone or with occasional assistance (e.g. “heavy work domestic help”) | 5 |
| *Don’t Know (Don’t Read out)* | *6* |
| *Refused (Don’t Read out)* | *R* |

1. **Do you do the laundry?**

| No, all laundry must be done by others | 1 |
| --- | --- |
| Yes, launders small items, rinse stocking etc. | 2 |
| Yes, does personal laundry completely | 3 |
| *Don’t Know (Don’t Read out)* | *4* |
| *Refused (Don’t Read out)* | *R* |

1. **How easily do you travel?**

| Does not travel at all | 1 |
| --- | --- |
| Travel limited to taxi or automobile with assistance of another | 2 |
| Travels on public transportation when accompanied by another | 3 |
| Arranges own travel via taxi, but does not otherwise use public transportation | 4 |
| Travels independently on public transport or drive my own car | 5 |
| *Don’t Know (Don’t Read out)* | *6* |
| *Refused (Don’t Read out)* | *R* |

1. **Are you responsible for your medication?**

| No, is not capable of dispensing own medication | 1 |
| --- | --- |
| Yes, takes responsibility if medication is prepared in advance in separate dosage | 2 |
| Yes, responsible for taking medication in correct dosages at correct times | 3 |
| *Don’t Know (Don’t Read out)* | *4* |
| *Refused (Don’t Read out)* | *R* |

1. **Are you responsible for your finances?**

| No, incapable of handling money | 1 |
| --- | --- |
| Yes, manages day-to-day purchases, but needs help with banking, major purchases, etc. | 2 |
| Yes, manages financial matters independently (budget, writes checks, pays rent, bills, goes to bank), collects and keeps tract of income | 3 |
| *Don’t Know (Don’t Read out)* | *4* |
| *Refused (Don’t Read out)* | *R* |

***Physical Activity***

*The next few questions ask about your levels of physical activity and exercise.*

**21A) During a typical 7-Day period (a week), how many times on average do you do STRENUOUS EXERCISE for more than 15 minutes during your free time?**

**By free time we mean when not working, or walking or cycling for transport.**

|  | **Times per week** |
| --- | --- |
| By STRENUOUS EXERCISE we mean activities like running, jogging, hockey, football, soccer, squash, basketball, cross country skiing, judo, roller skating, vigorous swimming, vigorous long distance bicycling | **_________________**  Code ‘888’ if don’t know  Code ‘999’ if refused |

**21B) During a typical 7-Day period (a week), how many times on average do you do MODERATE EXERCISE for more than 15 minutes during your free time?**

**By free time we mean when not working, or walking or cycling for transport.**

|  | **Times per week** |
| --- | --- |
| By MODERATE EXERCISE we mean activities like a fast walking, baseball, tennis, easy bicycling, volleyball, badminton, easy swimming or dancing | **_________________**  Code ‘888’ if don’t know  Code ‘999’ if refused |

**21C) During a typical 7-Day period (a week), how many times on average do you do MILD EXERCISE for more than 15 minutes during your free time?**

**By free time we mean when not working, or walking or cycling for transport.**

|  | **Times per week** |
| --- | --- |
| By MILD EXERCISE we mean activities like yoga, archery, fishing from river bank, bowling, golf, snow-mobiling, easy walking | **_________________**  Code ‘888’ if don’t know  Code ‘999’ if refused |

***Leisure:***

**22) During a typical 7-day period (a week), in your free time, how often do you engage in any regular activity long enough to work up a sweat (heart beats rapidly)?**

**By free time we mean when not working, or walking or cycling for transport.**

| Often | 1 |
| --- | --- |
| Sometimes | 2 |
| Never/rarely | 3 |
| *Don’t know* | *4* |
| *Refused* | *R* |

***Diet quality***

**23) Are you a vegetarian?**

| Yes | 1 |
| --- | --- |
| No | 2 |
| *Don’t know* | 3 |
| *Refused* | *R* |

*If YES: Please confirm that the person does not eat meat or any seafood (Pescatarian). If they say they eat meat/seafood sometimes select NO*

**17) How many times a week do you eat vegetables with your meal at night? Exclude hot chips.**

| 2 or less times per week | 1 |
| --- | --- |
| 3-4 times per week | 2 |
| 5 or more times per week | 3 |
| *Don’t know* | *4* |
| *Refused* | *R* |

**18) How many times a week do you usually eat a serving of these vegetables when they are in season?**

*As I read through the options can you let me know if you would have one or more serves per week?*

*On a usual week, would you eat at least one or more servings of the following vegetables when they are in season?*

Select all that apply

|  | **Less than once a week or never** | **Once a week or more often** |
| --- | --- | --- |
| Potato (boiled, mashed, baked) | 0 | 1 |
| Pumpkin | 0 | 1 |
| Sweet potato | 0 | 1 |
| Cauliflower | 0 | 1 |
| Green beans | 0 | 1 |
| Spinach | 0 | 1 |
| Cabbage or Brussel sprouts | 0 | 1 |
| Peas including frozen | 0 | 1 |
| Broccoli | 0 | 1 |
| Carrots | 0 | 1 |
| Zucchini, eggplant, squash | 0 | 1 |
| Capsicum | 0 | 1 |
| Corn, sweet corn, corn on the cob | 0 | 1 |
| Mushrooms | 0 | 1 |
| Tomatoes | 0 | 1 |
| Lettuce | 0 | 1 |
| Celery, cucumber | 0 | 1 |
| Avocado | 0 | 1 |
| Onion, spring onion leek | 0 | 1 |
| *None of the above* | *20* | |
| *Don’t know* | *21* | |
| *Refused* | *R* | |

**19) How many pieces of fruit do you usually eat per day? Include all types**

| 1 or less per day | 1 |
| --- | --- |
| 2 or more per day | 2 |
| *Don’t know* | *3* |
| *Refused* | *R* |

**20) How many times a week do you usually eat a serving of these fruits when they are in season? One serving can be one medium piece, two small pieces, one cup of canned fruit or one heaped tablespoon of dried fruit.**

*As I read through the options can you let me know again if you would have one or more serves per week?*

*On a usual week, would you eat at least one or more servings of these fruits when they are in season?*

*One serving can be one medium piece, two small pieces, one cup of canned fruit or one heaped teaspoon of dried fruit.*

Select all that apply

|  | **Less than once a week or never** | **Once a week or more often** |
| --- | --- | --- |
| Canned fruit, e.g. peaches, Two fruits | 0 | 1 |
| Fresh fruit salad | 0 | 1 |
| Dried fruit, e.g. sultanas, dried apricots | 0 | 1 |
| Apple or pear | 0 | 1 |
| Orange, mandarin, grapefruit | 0 | 1 |
| Banana | 0 | 1 |
| Peach, nectarine, plum or apricot | 0 | 1 |
| Mango or paw-paw | 0 | 1 |
| Pineapple | 0 | 1 |
| Grapes, strawberries, blueberries | 0 | 1 |
| Melon, e.g. watermelon, rockmelon, honeydew melon | 0 | 1 |
| *None of the above* |  | *12* |
| *Don’t know* |  | *13* |
| *Refused* |  | *R* |

**21) How many times a week do you usually eat a serving of these protein foods?**

*As I read through the options again can you let me know if they apply to you.*

*On a usual week, would you eat at least one or more servings of these protein foods?*

Select all that apply

|  | **Less than once a week or never** | **Once a week or more often** |
| --- | --- | --- |
| Mince dish, e.g. Spaghetti Bolognese, rissoles, lasagne | 0 | 1 |
| Meat (beef or lamb), e.g. roast, chops, steak, strips | 0 | 1 |
| Chicken, e.g. BBQ, breast, thigh, schnitzel | 0 | 1 |
| Pork, e.g. roast, chops, strips | 0 | 1 |
| Fresh fish not crumbed or battered | 0 | 1 |
| Canned tuna, salmon, sardines including patties | 0 | 1 |
| Other seafood, e.g. prawns, lobster | 0 | 1 |
| *None of the above* |  | *8* |
| *Don’t know* |  | *9* |
| *Refused* |  | *R* |

**22) How many times a week do you usually eat a serving of these protein foods?**

*As I read through the options, can you let me know if they apply to you.*

*On a usual week, would you eat at least one or more servings of these protein foods?*

Select all that apply

|  | **Less than once a week or never** | **Once a week or more often** |
| --- | --- | --- |
| Nuts, e.g. peanuts, almonds | 0 | 1 |
| Peanut butter, Nutella | 0 | 1 |
| Eggs, e.g. boiled, scrambled | 0 | 1 |
| Soybeans, tofu | 0 | 1 |
| Baked beans | 0 | 1 |
| Other beans, lentils, e.g. chickpeas, split peas | 0 | 1 |
| *None of the above* |  | *7* |
| *Don’t know* |  | *8* |
| *Refused* |  | *R* |

**23) What type of bread do you usually eat?**

| Brown (multigrain, wholemeal) | 1 |
| --- | --- |
| White | 2 |
| Other | 3 |
| Don't eat Bread | 4 |
| *Don’t know* | *5* |
| *Refused* | *R* |

**24) How many times a week do you usually eat a serving/bowl/slice of these grains?**

*As I read through the options, can you let me know if they apply to you.*

*On a usual week, would you eat at least one or more servings of these grains?*

*E.g. (bowl or slice)*

Select all that apply

|  | **Less than once a week or never** | **Once a week or more often** |
| --- | --- | --- |
| Muesli | 0 | 1 |
| Porridge | 0 | 1 |
| Breakfast cereal, e.g. Weet-bix, Nutri-grain, Cornflakes, Sultana Bran | 0 | 1 |
| Bread, pita bread, roll or toast all types | 0 | 1 |
| English muffin, bagel or crumpet | 0 | 1 |
| Rice | 0 | 1 |
| Other grains, e.g. cous cous, burghul, quinoa | 0 | 1 |
| Noodles, e.g. egg noodles (yellow), rice noodles (white) | 0 | 1 |
| Pasta, e.g. spaghetti, lasagne, pasta bake | 0 | 1 |
| Clear soup with rice or noodles | 0 | 1 |
| Tacos, burritos, enchiladas | 0 | 1 |
| *None of the above* |  | *12* |
| *Don’t know* |  | *13* |
| *Refused* |  | *R* |

**25) Add up how many times a day you have a glass of milk, a tub of yoghurt or a slice of cheese**

| 1 or less times per day | 1 |
| --- | --- |
| 2 or more times per day | 2 |
| *Don’t know* | *3* |
| *Refused* | *R* |

**26) What type of milk do you usually drink?**

| Reduced fat milk, skim milk or soy milk | 1 |
| --- | --- |
| Full cream milk, rice milk or other milk | 2 |
| Don’t drink milk | 3 |
| *Don’t know* | *4* |
| *Refused* | *R* |

**27) How many times do you usually eat/drink a serving/glass/slice of these dairy foods?**

*As I read through the options, can you let me know if they apply to you.*

*On a usual week, would you eat or drink at least one or more servings of these dairy foods?*

*E.g. (glass or slice)*

Select all that apply

|  | **Less than once a week or never** | **Once a week or more often** |
| --- | --- | --- |
| Flavoured milk, e.g. hot chocolate, milkshake, smoothie | 0 | 1 |
| Plain milk- glass or with cereal | 0 | 1 |
| Ice cream- vanilla or flavoured, sundaes, cones | 0 | 1 |
| Frozen yoghurt | 0 | 1 |
| Yoghurt (not frozen) plain or flavoured | 0 | 1 |
| Cottage cheese or ricotta | 0 | 1 |
| Cheese, including cheese on sandwiches, biscuits or on toast | 0 | 1 |
| Cheese spread, cream cheese | 0 | 1 |
| *None of the above* |  | *9* |
| *Don’t know* |  | *10* |
| *Refused* |  | *R* |

**28) How many glasses of water a day do you usually drink? Water including bottled water, unflavoured mineral water, tap water.**

| 3 or less per day | 1 |
| --- | --- |
| 4 or more per day | 2 |
| *Don’t know* | *3* |
| *Refused* | *R* |

**29) How many times a week do you usually eat a serving of these sauces and spreads?**

*As I read through the options, can you let me know if they apply to you.*

*On a usual week, would you eat or drink at least one or more servings of these sauces and spreads?*

Select all that apply

|  | **Less than once a week or never** | **Once a week or more often** |
| --- | --- | --- |
| Vegemite, Mighty Mite, Promite, Marmite | 0 | 1 |
| Tomato or BBQ sauce, tomato paste or tomato based sauces | 0 | 1 |
| *None of the above* |  | *3* |
| *Don’t know* |  | *4* |
| *Refused* |  | *R* |

**13) How often do you add salt to your cooking?**

| Never/rarely | 1 |
| --- | --- |
| Sometimes | 2 |
| Usually | 3 |
| Always | 4 |
| *Don’t know* | *6* |
| *Refused* | *R* |

**13) How often do you use salt at the table?**

| Never/rarely | 1 |
| --- | --- |
| Sometimes | 2 |
| Usually | 3 |
| Always | 4 |
| *Don’t know* | *6* |
| *Refused* | *R* |

***Carer***

*The next few questions are about any help you may receive from an informal carer, that is the main person who does not receive payment to assist you in your daily living tasks, and is not your doctor or other health professional.*

**7) Who is your main carer at home?**

*By main carer we mean the person who most helps you with daily living tasks at home, and not your doctor or other health professional.*

| Partner/spouse | 1 |  |
| --- | --- | --- |
| Child | 2 |  |
| Parent | 3 |  |
| Other family member | 4 |  |
| Friend | 5 |  |
| Neighbour | 6 |  |
| I do not have a carer at home with me | 7 |  |
| Other (please specify) | 8 | Go to Q8 |
| Live in fulltime care at a facility | 9 |  |
| *Don’t know* | *10* |  |
| *Refused* | *R* |  |

**7) Who is your main carer at home?**

**[open response to “Other (please specify)” above]**

**_____________________________________________________________**

***Income***

**47) What is your total personal gross income from all sources?**

| $1 to $199 per week | 1 |
| --- | --- |
| $200 to $299 per week | 2 |
| $300 to $399 per week | 3 |
| $400 to $599 per week | 4 |
| $600 to $799 per week | 5 |
| $800 to $999 per week | 6 |
| $1,000 to $1,249 per week | 7 |
| $1,250 to $1,499 per week | 8 |
| $1,500 to $1,999 per week | 9 |
| $2,000 or more per week | 10 |
| Nil income | 11 |
| Negative income | 12 |
| *Don’t Know (Don’t Read out)* | *13* |
| *Refused (Don’t Read out)* | *R* |

***Health Services***

*The final section of this survey asks about the health services that you may have received, including hospital admissions, GP visits, specialist appointments, medications, etc.*

**35) How much time, in total, did you spend in long-stay hospital?**

**Days Months Years**

(max 30) (max 11) (max 20)

| *Don’t Know (Don’t Read out)* | *4* |
| --- | --- |
| *Refused (Don’t Read out)* | *R* |

***Services/Medication***

*The following questions will ask about any health services or treatment you have used in recent months. These can be related or unrelated to your stroke/TIA event.*

**48) Have you used any of the following INPATIENT services in the last 6 months?**

| **Service** | **Name of facility** | **Number of admissions** | **Total number of inpatient days)** |
| --- | --- | --- | --- |
| Acute hospital ward (short stay unit, often an admission of less than 24hrs) |  |  |  |
| Rehabilitation ward/facility |  |  |  |
| General medical ward (e.g. Cardiac, Respiratory, Endocrine, Gastroenterology or trauma, often an overnight admission) |  |  |  |
| Other (Please specify) |  |  |  |
| *Don’t know* |  |  |  |
| *Refused* |  |  |  |

**49) Have you used any of the following OUTPATIENT services in the last 3 months?** An outpatient service refers to those which do not require an overnight hospital admission.

| **Service** | **Name of facility** | **Unit of measurement (appointment, day)** | **No. of units received** |
| --- | --- | --- | --- |
| Psychiatric outpatient visit |  |  |  |
| Other hospital outpatient visit |  |  |  |
| Outpatient rehabilitation team |  |  |  |
| Community stroke team |  |  |  |
| Other (please specify) |  |  |  |
| *Don’t know (don’t read out)* |  |  |  |
| *Refused (don’t read out)* |  |  |  |

**50) Have you used any of the following health care services over the last 3 months?** *Excluding contact with the professional and team members noted above in the last few questions and any services provided by staff in the accommodation facility.*

| **Service** | **Total number of contacts** | **Average contact time (hours)** |
| --- | --- | --- |
| 1 Occupational therapist |  |  |
| 2 Physiotherapist |  |  |
| 3 Speech therapist |  |  |
| 4 Dietician |  |  |
| 5 Psychologist |  |  |
| 6 Community psychiatric nurse |  |  |
| 7 Other nursing services |  |  |
| 8 Social worker |  |  |
| 9 Neuropsychologist |  |  |
| 10 Geriatrician |  |  |
| 11 Neurologist |  |  |
| 12 Rehabilitation physician |  |  |
| 13 Podiatrist |  |  |
| 14 Individual counselling/therapy |  |  |
| 15 Group counselling/therapy |  |  |
| 16 Home help/home care worker |  |  |
| 17 Outreach worker/family support |  |  |
| 18 General practitioner (aka GP, local doctor, family doctor) |  |  |
| 19 Dentist |  |  |
| 20 Optometrist |  |  |
| 21 Ophthalmologist |  |  |
| 22 Other specialist(Please specify) |  |  |
| -23 Don’t Know (Don’t Read out) |  |  |
| .R Refused (Don’t Read out) |  |  |

**51) Were there any other medications, not provided by a doctor, which you took for health management following your stroke? (such as Aspirin, ibuprofen, etc.).**

| **Name of drug** | **Dosage (e.g. ml., grms, number of tablets)** | **Dosage frequency** |
| --- | --- | --- |
| 1. |  |  |
| 2. |  |  |
| 3. |  |  |
| 4. |  |  |
| 5. |  |  |

**Thank you for completing this survey. You will be contacted again by the research team within a week to notify you of your group allocation. We would like to thank you on behalf of the research team for taking part in this study.** **We look forward to contacting you in a few months for your final Prevent Second Stroke survey.**
